# Supplementary material for: Gut microbiota carcinogen metabolism causes distal tissue tumours
Source: Nature. 2024 Jul 31;632(8027):1137–44. doi: 10.1038/s41586-024-07754-w (PMC11358042; doi:10.1038/s41586-024-07754-w)
Supplement: Supplementary file 1 — All histology images of mouse bladders after 20 weeks of BBN exposure. [file 41586_2024_7754_MOESM1_ESM.pdf]

nature portfolio

https://doi.org/10.1038/s41586-024-07754-w

Supplementary information

Gut microbiota carcinogen metabolism causes distal tissue tumours

In the format provided by the authors and unedited

Supplementary Data 1.
All bladder histology images used for the quantification of cancer stage per mouse summarize in Fig. 1C

Group	Treatment	No. of animals	Normal	CIS	Invasion	Sarcomatoid carcinoma
I	BBN/ABX	6	5	1		
	BBN	5			5	
II	BBN/ABX	9	8			1
	BBN	7	3	1	3	
III	BBN/ABX	5	2	1	2	
	BBN	5		2	2	1
IV	BBN/ABX	4	3		1	
	BBN	5	1	2	2	
V	BBN/ABX	8	8			
	BBN	8	3	2	3	

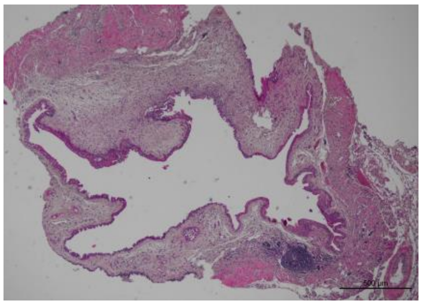
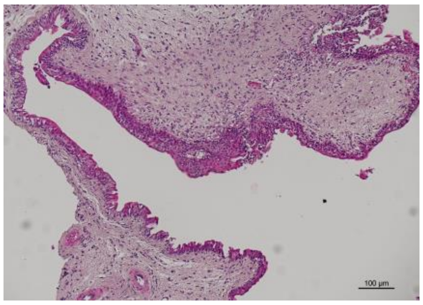

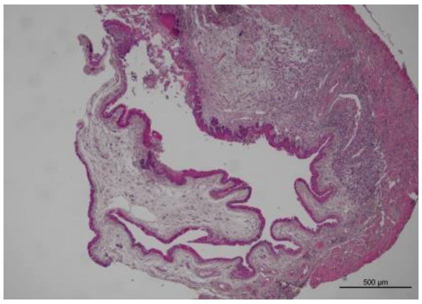
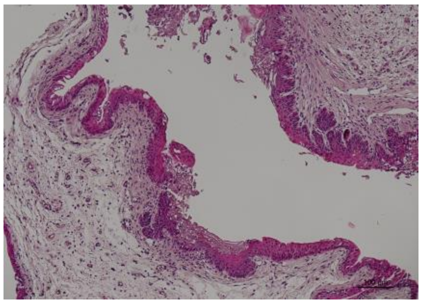

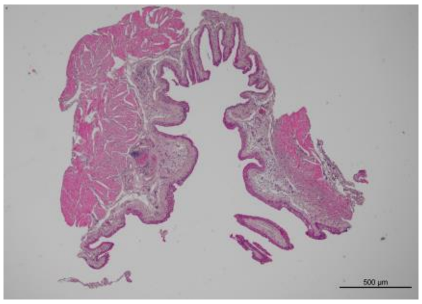
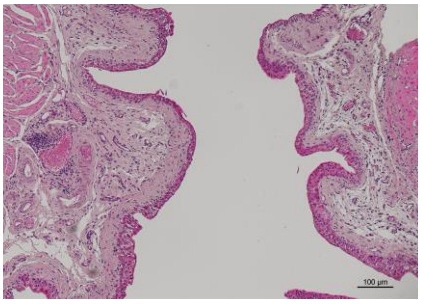

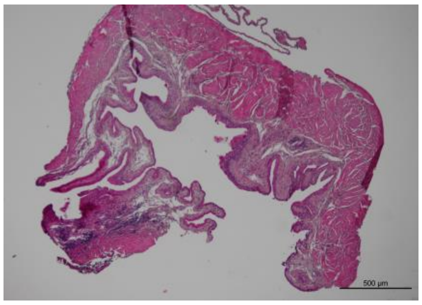
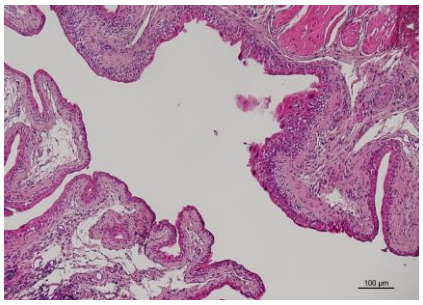

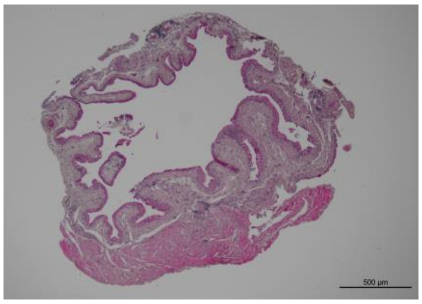
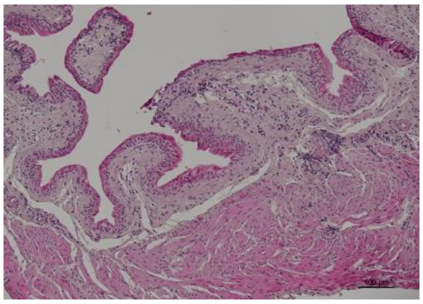

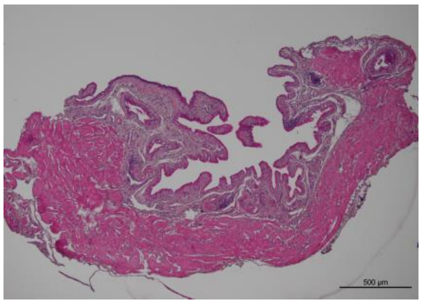
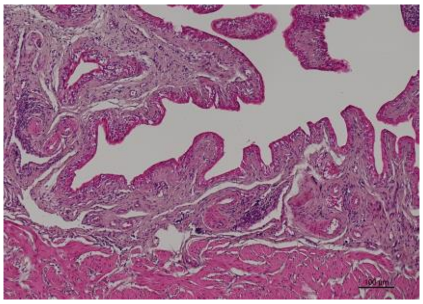

Group I	BBN/ABX	Normal
Group I	BBN/ABX	CIS
Group I	BBN/ABX	Normal
Group I	BBN/ABX	Normal
Group I	BBN/ABX	Normal
Group I	BBN/ABX	Normal

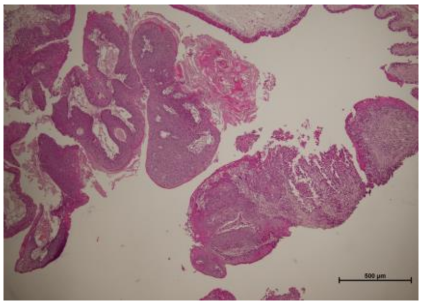	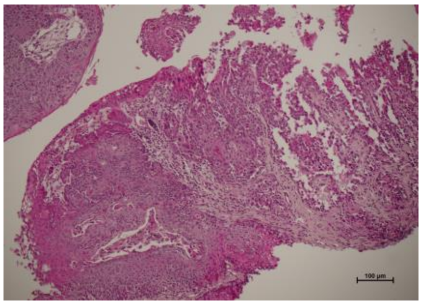	Group I	BBN	Invasion
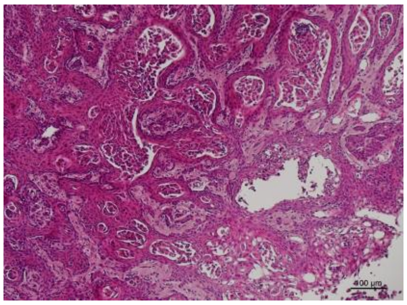	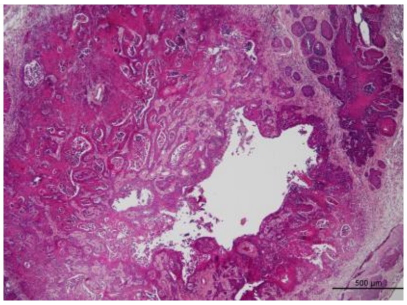	Group I	BBN	Invasion
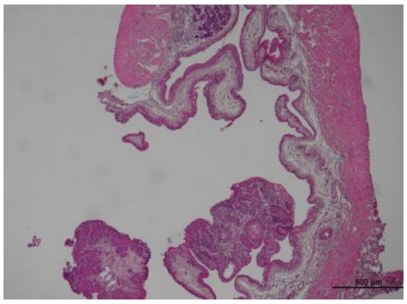	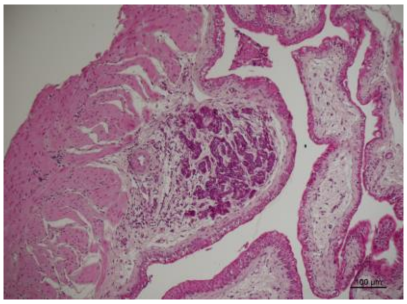	Group I	BBN	Invasion
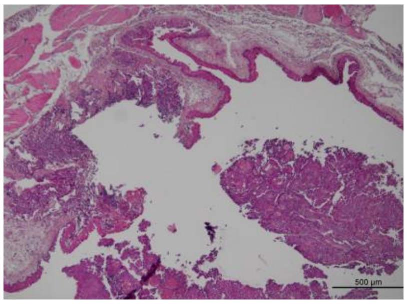	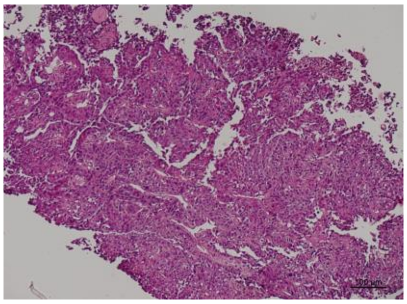	Group I	BBN	Invasion
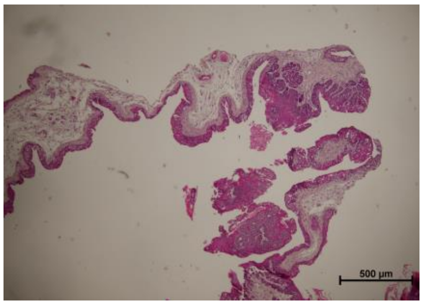	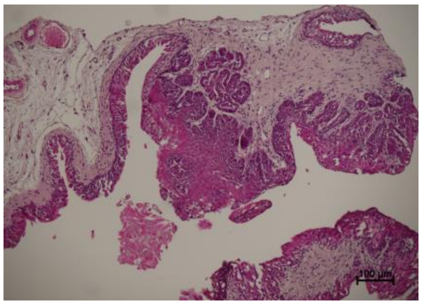	Group I	BBN	Invasion

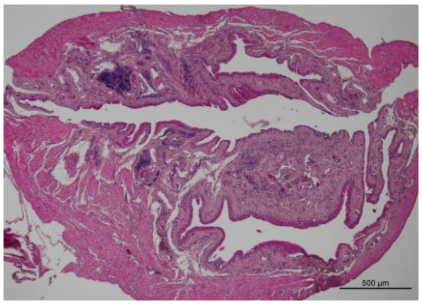	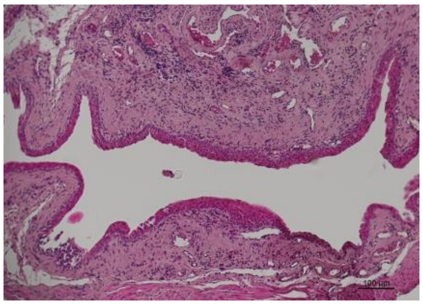	Group II	BBN/ABX	Normal
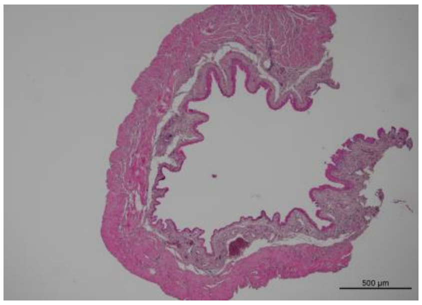	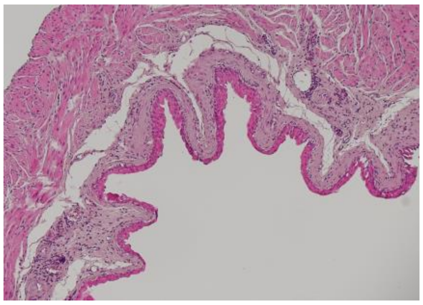	Group II	BBN/ABX	Normal
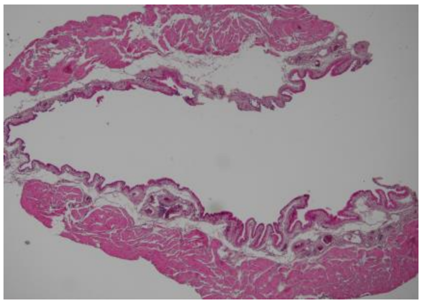	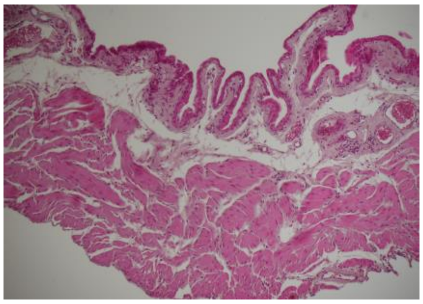	Group II	BBN/ABX	Normal
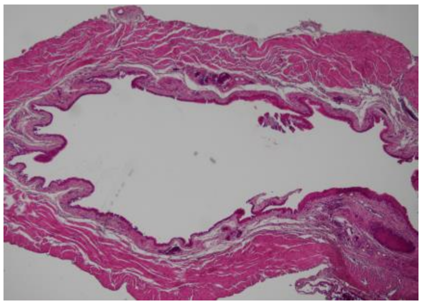	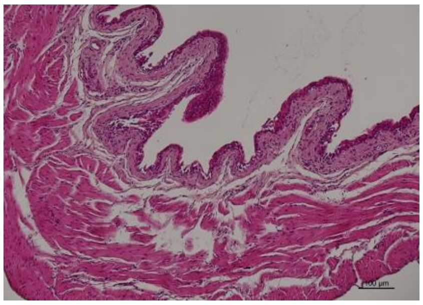	Group II	BBN/ABX	Normal
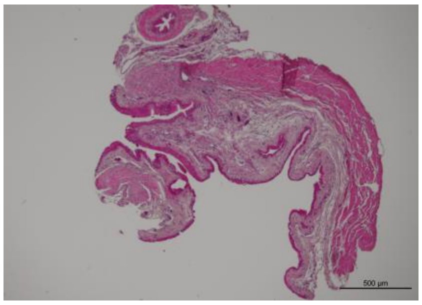	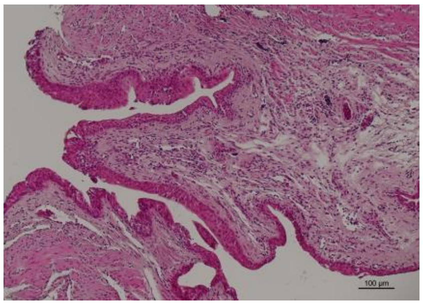	Group II	BBN/ABX	Normal

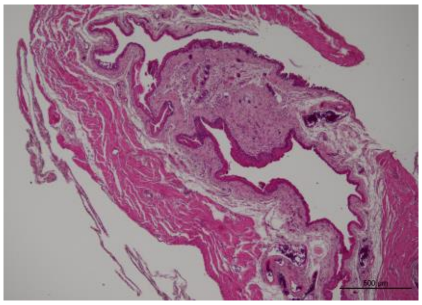	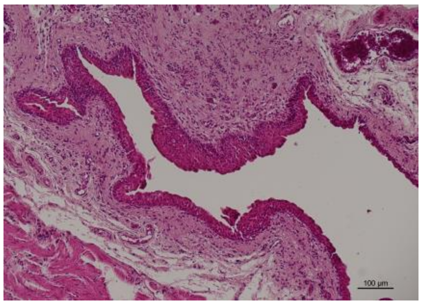	Group II	BBN/ABX	Normal
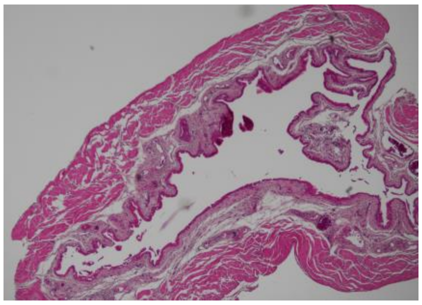	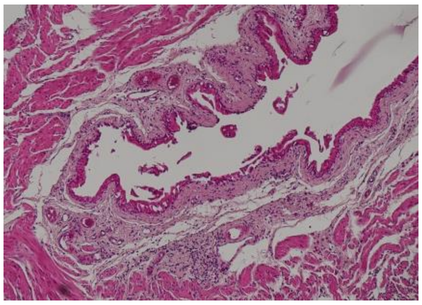	Group II	BBN/ABX	Normal
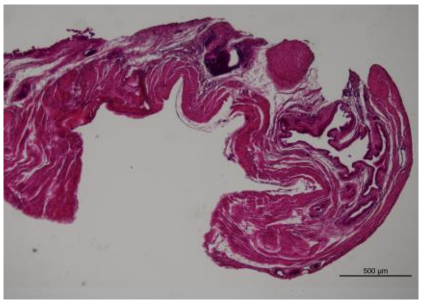	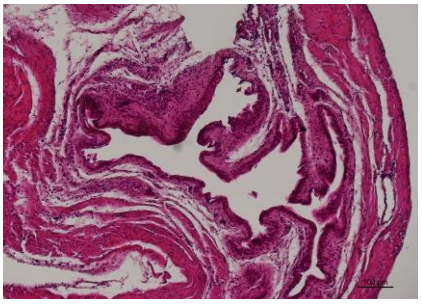	Group II	BBN/ABX	Normal
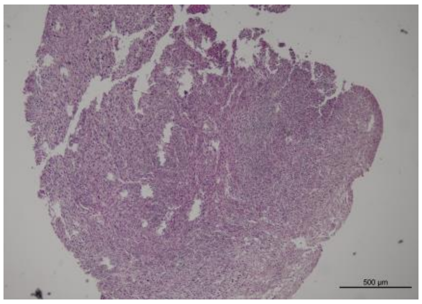	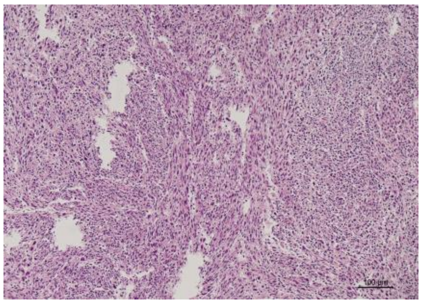	Group II	BBN/ABX	Sarcomatoid carcinoma

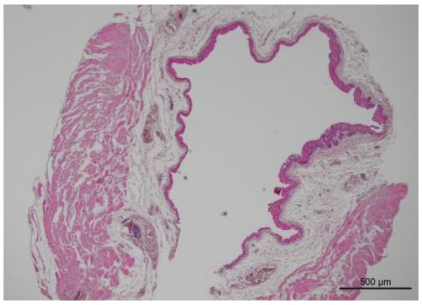	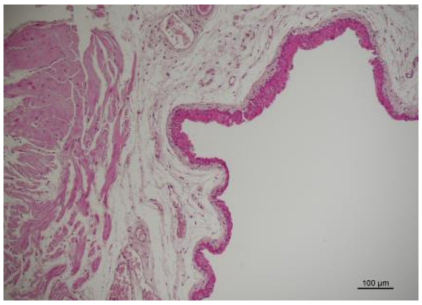	Group II	BBN	Normal
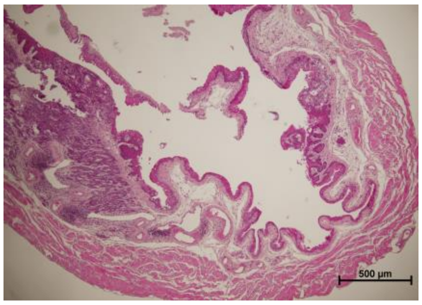	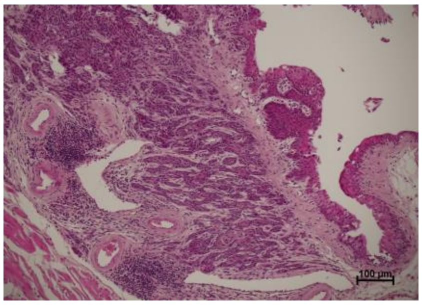	Group II	BBN	Invasion
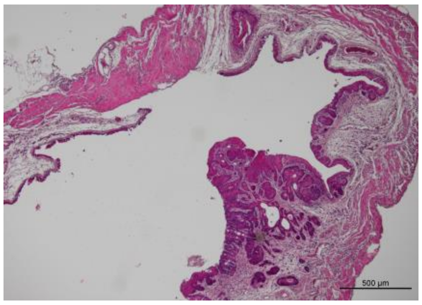	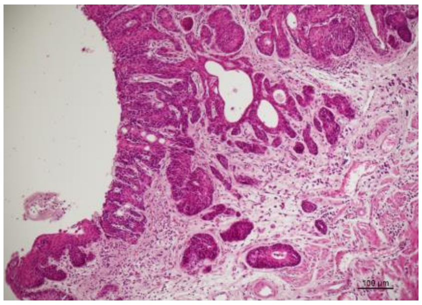	Group II	BBN	Invasion
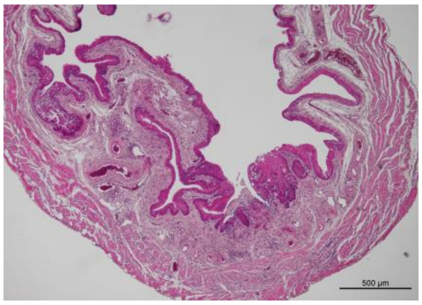	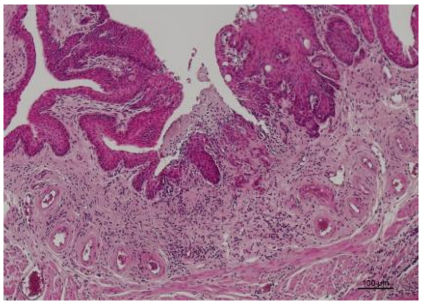	Group II	BBN	CIS
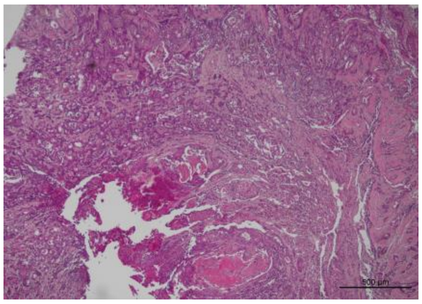	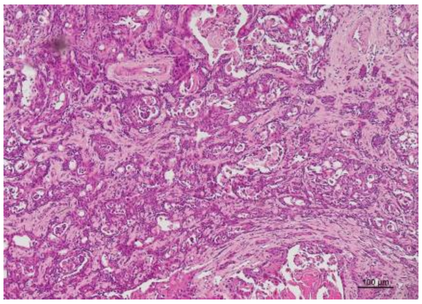	Group II	BBN	Invasion
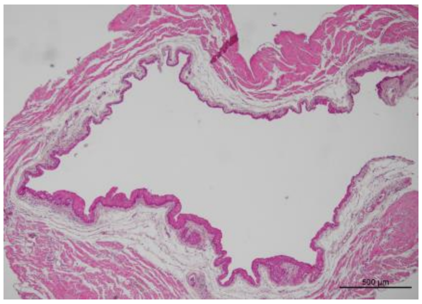	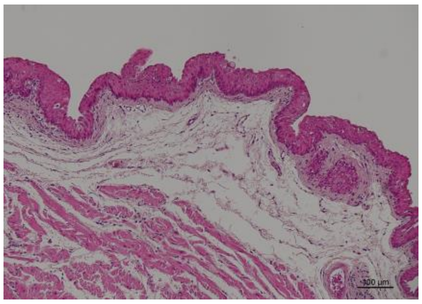	Group II	BBN	Normal
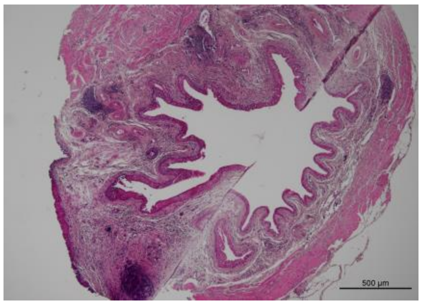	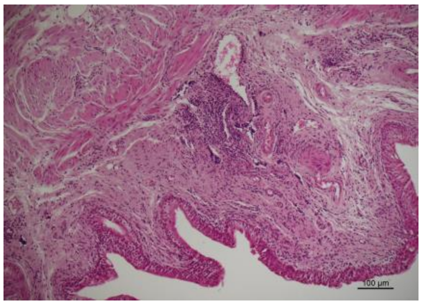	Group II	BBN	Normal

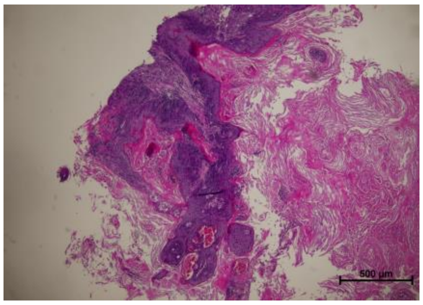	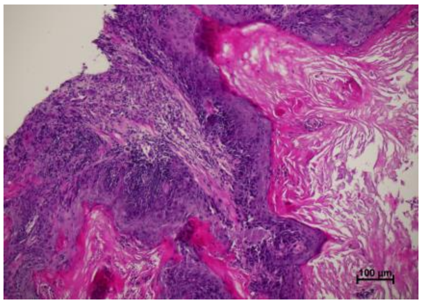	Group III	BBN/ABX	Invasion
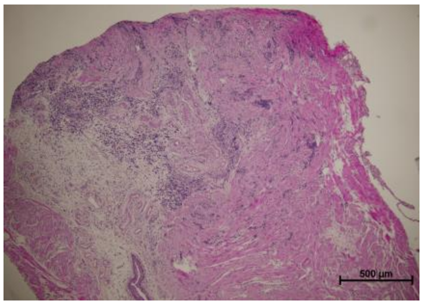	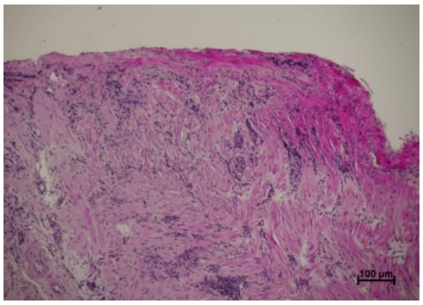	Group III	BBN/ABX	Invasion
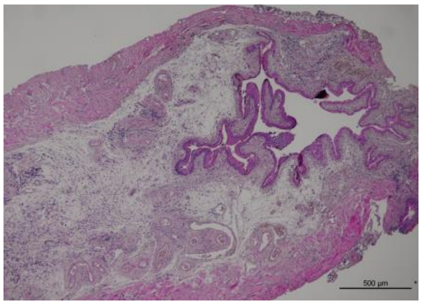	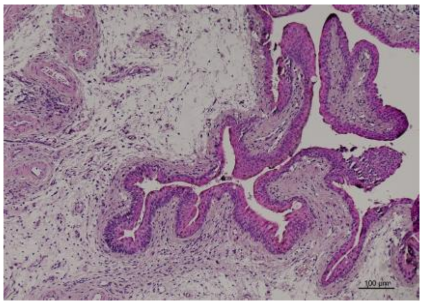	Group III	BBN/ABX	Normal
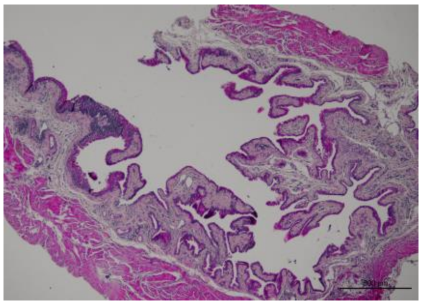	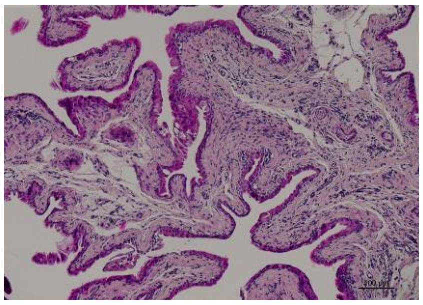	Group III	BBN/ABX	Normal
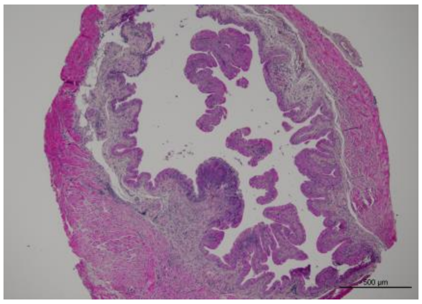	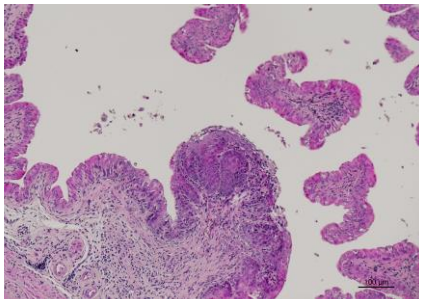	Group III	BBN/ABX	CIS

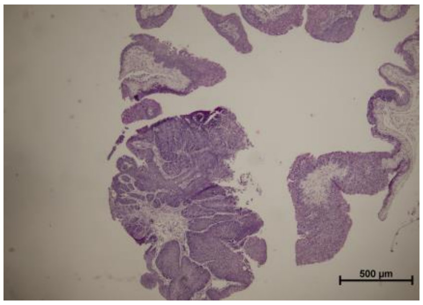	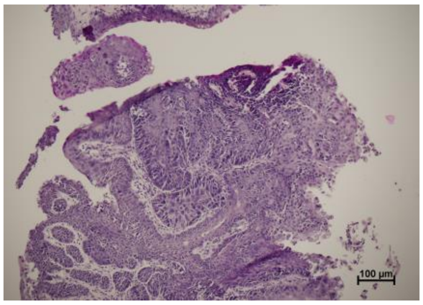	Group III	BBN	Invasion
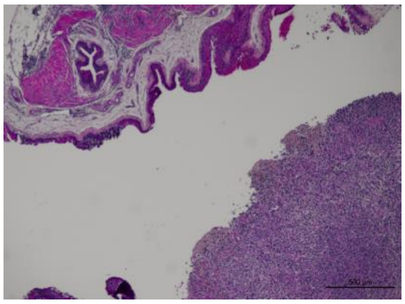	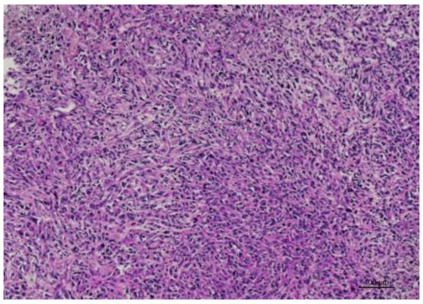	Group III	BBN	Sarcomatoid carcinoma
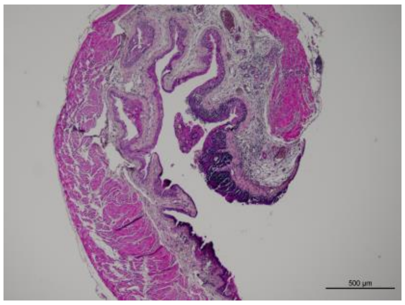	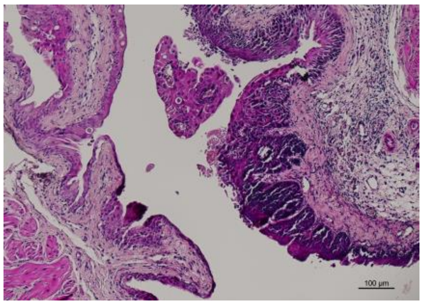	Group III	BBN	CIS
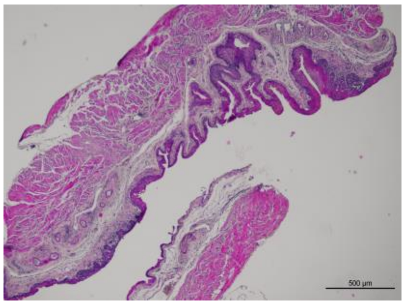	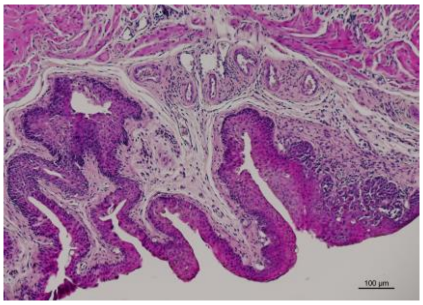	Group III	BBN	CIS
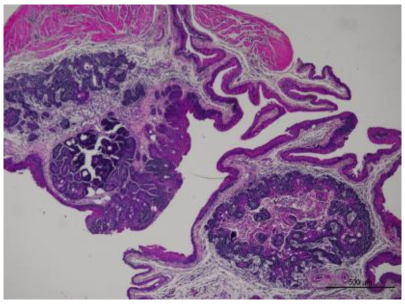	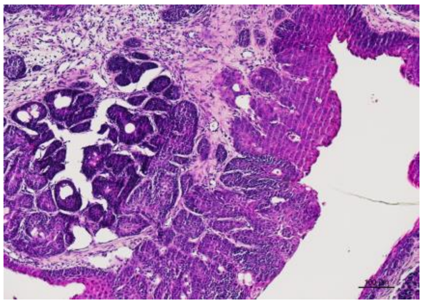	Group III	BBN	Invasion

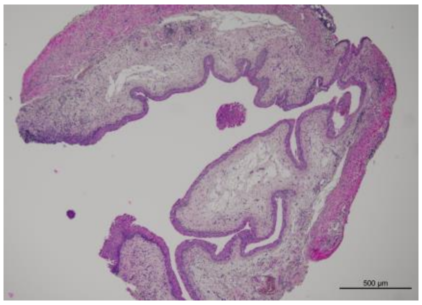	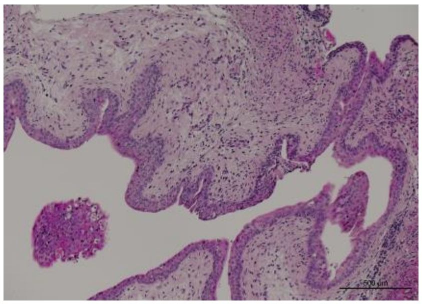	Group IV	BBN/ABX	Normal
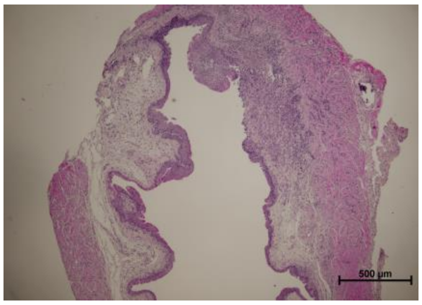	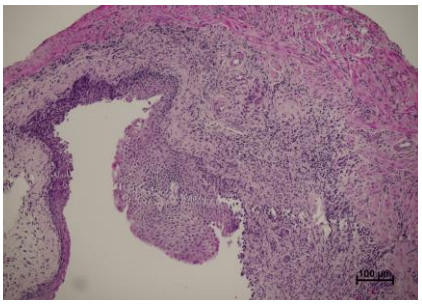	Group IV	BBN/ABX	Invasion
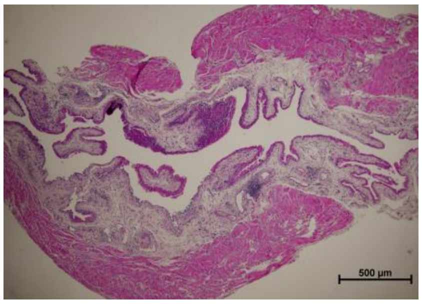	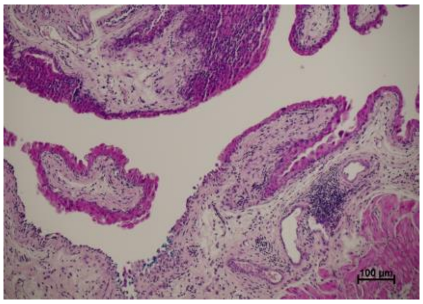	Group IV	BBN/ABX	Normal
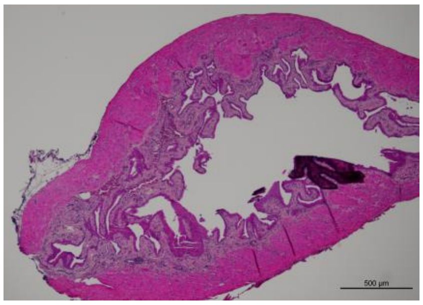	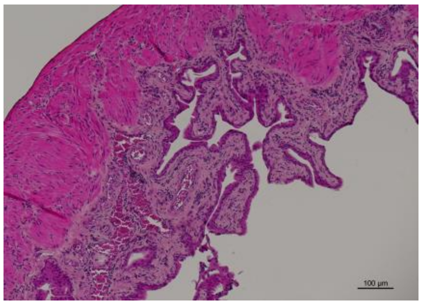	Group IV	BBN/ABX	Normal

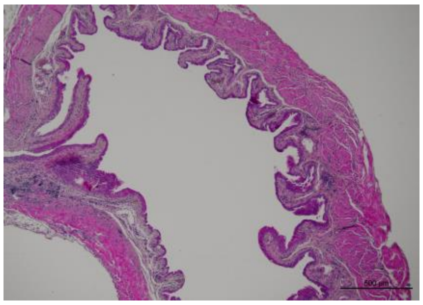
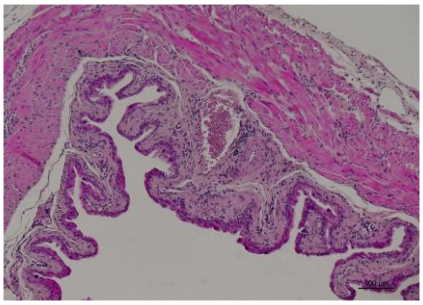
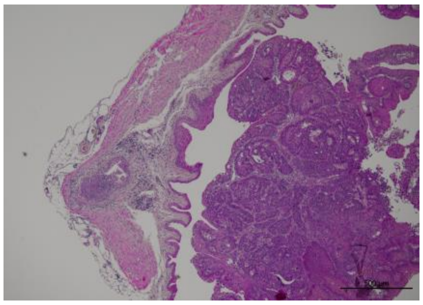
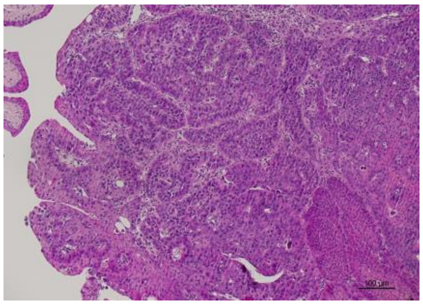
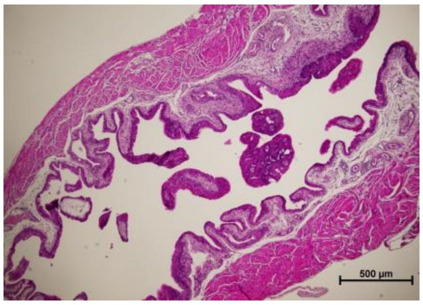
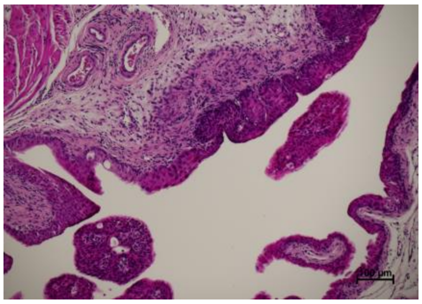
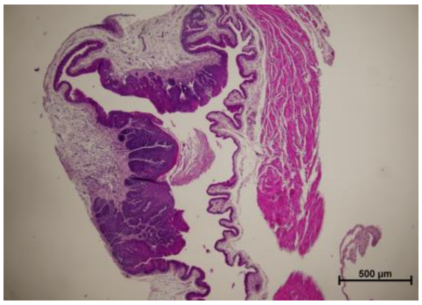
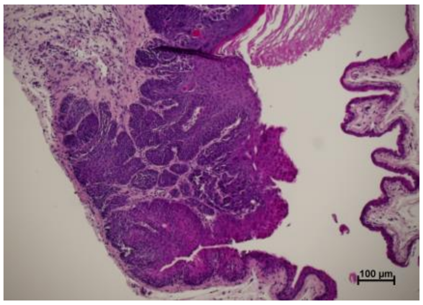
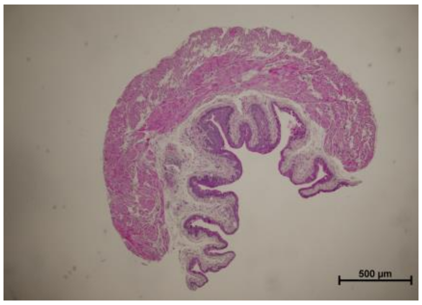
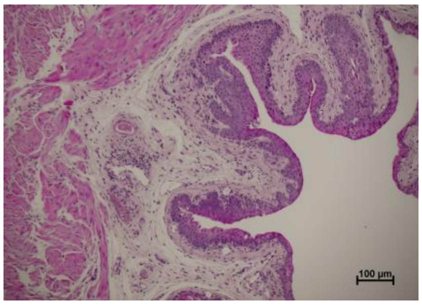

Group IV	BBN	Normal
Group IV	BBN	Invasion
Group IV	BBN	CIS
Group IV	BBN	Invasion
Group IV	BBN	CIS

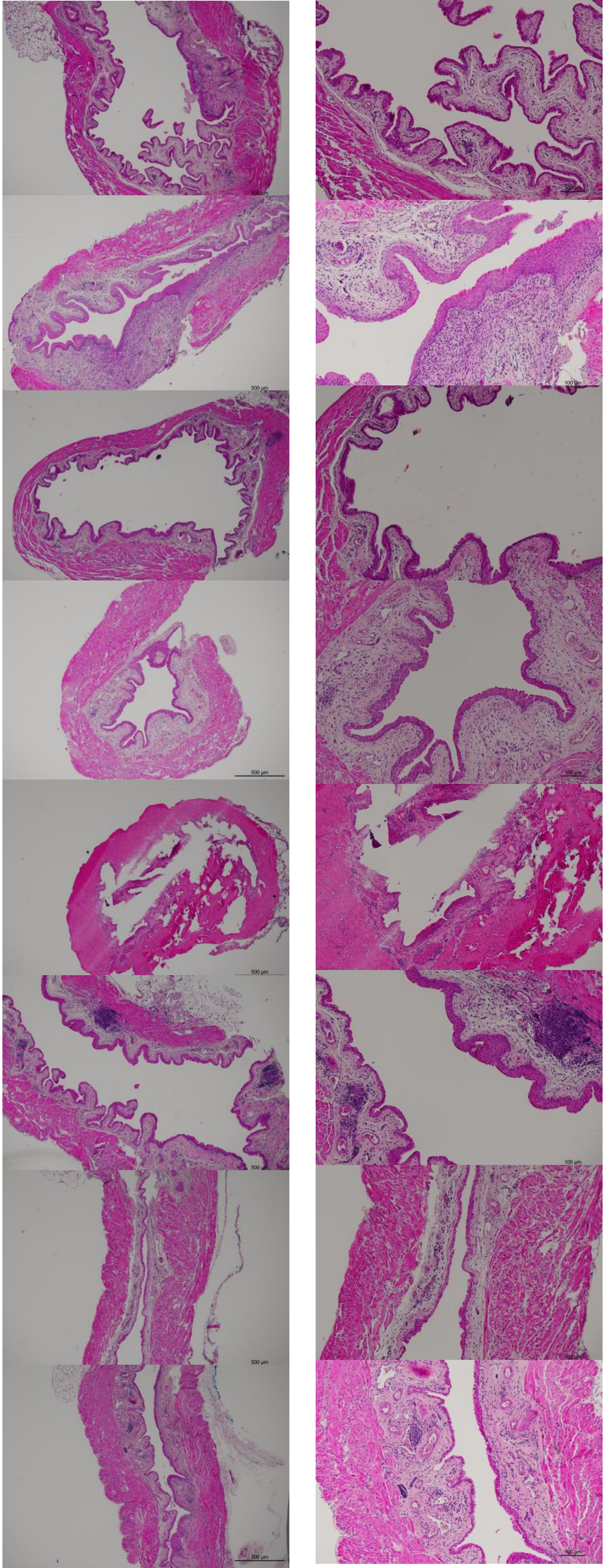

Group V	BBN/ABX	Normal
Group V	BBN/ABX	Normal
Group V	BBN/ABX	Normal
Group V	BBN/ABX	Normal
Group V	BBN/ABX	Normal
Group V	BBN/ABX	Normal
Group V	BBN/ABX	Normal
Group V	BBN/ABX	Normal

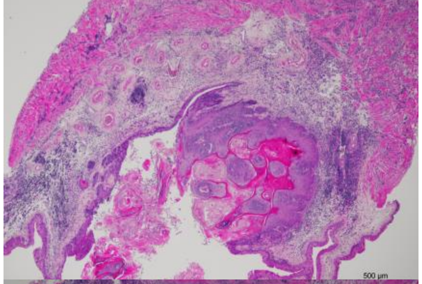	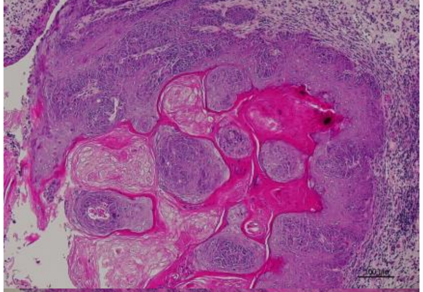	Group V	BBN	Invasion
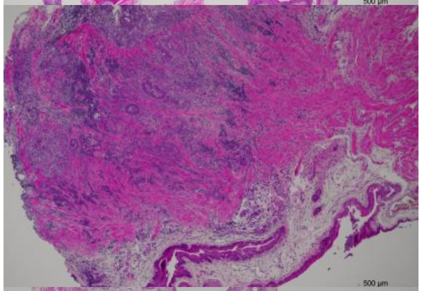	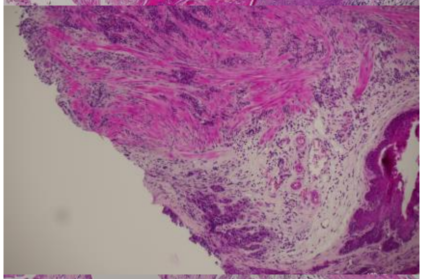	Group V	BBN	Invasion
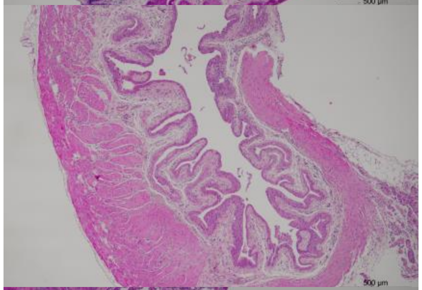	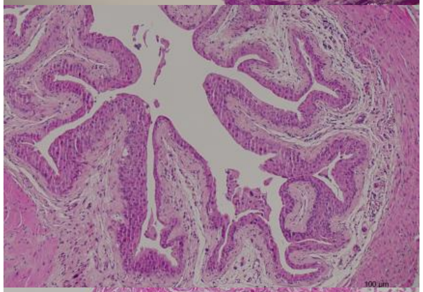	Group V	BBN	Normal
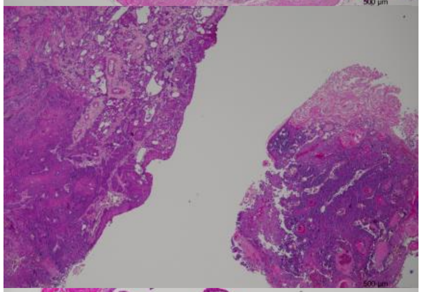	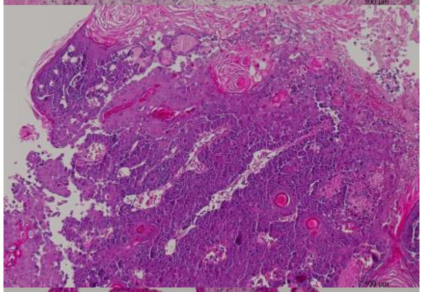	Group V	BBN	Invasion
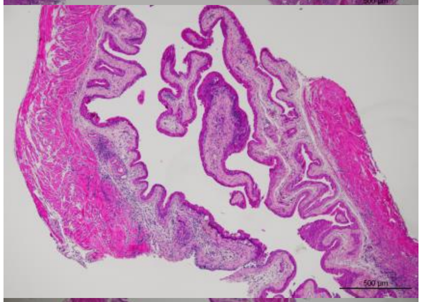	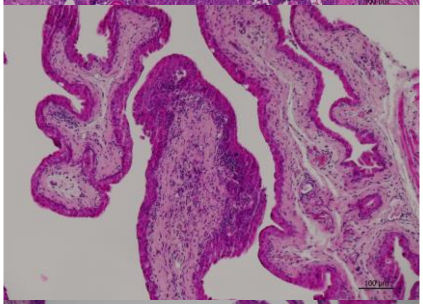	Group V	BBN	CIS
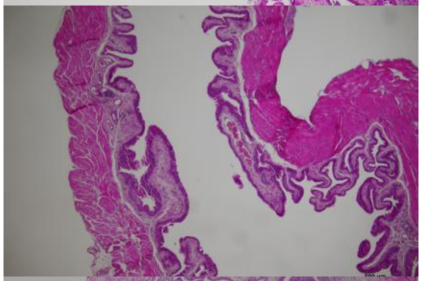	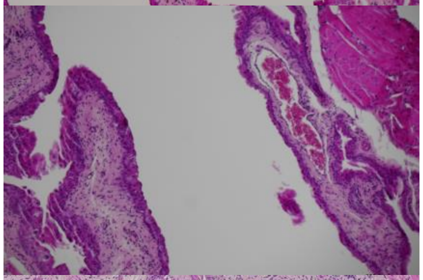	Group V	BBN	Normal
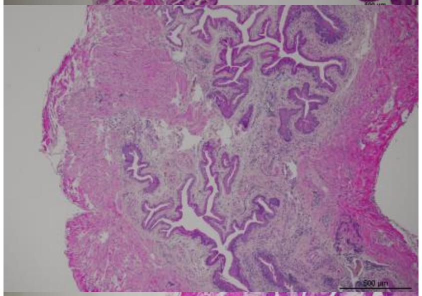	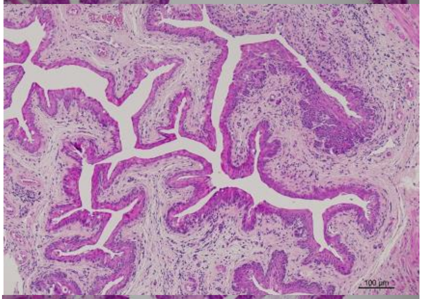	Group V	BBN	CIS
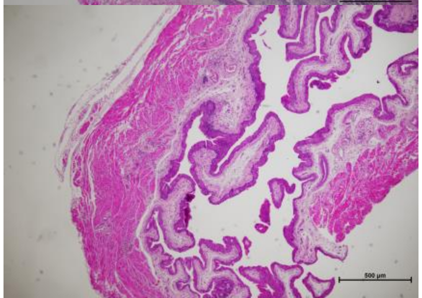	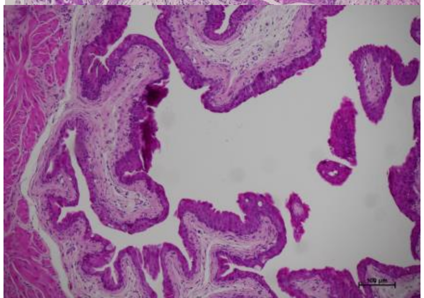	Group V	BBN	Normal
