# Supplementary material for: Gut microbiota carcinogen metabolism causes distal tissue tumours
Source: Nature. 2024 Jul 31;632(8027):1137–44. doi: 10.1038/s41586-024-07754-w (PMC11358042; doi:10.1038/s41586-024-07754-w)
Supplement: Supplementary file 2 — Reporting Summary [file 41586_2024_7754_MOESM2_ESM.pdf]

nature portfolio

Corresponding author(s): Michael Zimmermann PhD
Janos Terzic MD PhD

Last updated by author(s): Jun 6, 2024

Reporting Summary

Nature Portfolio wishes to improve the reproducibility of the work that we publish. This form provides structure for consistency and transparency in reporting. For further information on Nature Portfolio policies, see our Editorial Policies and the Editorial Policy Checklist.

Statistics

For all statistical analyses, confirm that the following items are present in the figure legend, table legend, main text, or Methods section.

n/a	Confirmed	
☐	☒	The exact sample size (*n*) for each experimental group/condition, given as a discrete number and unit of measurement
☐	☒	A statement on whether measurements were taken from distinct samples or whether the same sample was measured repeatedly
☐	☒	The statistical test(s) used AND whether they are one- or two-sided<br>*Only common tests should be described solely by name; describe more complex techniques in the Methods section.*
☒	☐	A description of all covariates tested
☐	☒	A description of any assumptions or corrections, such as tests of normality and adjustment for multiple comparisons
☐	☒	A full description of the statistical parameters including central tendency (e.g. means) or other basic estimates (e.g. regression coefficient) AND variation (e.g. standard deviation) or associated estimates of uncertainty (e.g. confidence intervals)
☐	☒	For null hypothesis testing, the test statistic (e.g. *F*, *t*, *r*) with confidence intervals, effect sizes, degrees of freedom and *P* value noted<br>*Give P values as exact values whenever suitable.*
☒	☐	For Bayesian analysis, information on the choice of priors and Markov chain Monte Carlo settings
☒	☐	For hierarchical and complex designs, identification of the appropriate level for tests and full reporting of outcomes
☒	☐	Estimates of effect sizes (e.g. Cohen's *d*, Pearson's *r*), indicating how they were calculated

Our web collection on statistics for biologists contains articles on many of the points above.

Software and code

Policy information about availability of computer code

Data collection	MassHunter Qualitative Analysis Software (Agilent Technologies, version 10.0) was used to acquire LC-MS data. NIS-Elements Viewer 4.600 was used to acquire histological images.
Data analysis	MassHunter Qualitative Analysis Software (Agilent Technologies, version 10.0) was used to determine retention time for all compounds to enable targeted analysis and quantification. Peak integration was carried out using MassHunter Quantitative Analysis Software (Agilent Technologies, version 10.0).The following open access software tools and packages were used for the analysis of 16s rRNA data: qiime 2020.8, FastQC v0.11.5, cutadapt 2.10, dada2, vsearch 2.7.0, mafft 7.471, fasttree 2.1.11, iTOL v 6.0. Statistical analysis was performed with R 4.0.0. The following open access software tools and packages were used for the analysis of whole genome sequences: FastQC v0.11.93, MultiQC 1.12, Trimmomatic 0.39, Spades v3.15.3, QUAST v5.0.2, kraken v2.0.74, gtdbtk v2.1.1, mTAGs v1.0.4, Prokka v1.13, Roary v3.7.0, Phandango v1.3.0, iTOL v6.

For manuscripts utilizing custom algorithms or software that are central to the research but not yet described in published literature, software must be made available to editors and reviewers. We strongly encourage code deposition in a community repository (e.g. GitHub). See the Nature Portfolio guidelines for submitting code & software for further information.

Data

Policy information about availability of data

All manuscripts must include a data availability statement. This statement should provide the following information, where applicable:

- Accession codes, unique identifiers, or web links for publicly available datasets
- A description of any restrictions on data availability
- For clinical datasets or third party data, please ensure that the statement adheres to our policy

Raw sequencing data have been deposited on the ENA server, with accession number PRJEB43561 and raw data of the LC-MS analysis have been deposited in the Metabolights public repository with the accession number MTBLS3581. The RefSeq dataset of curated 16S ribosomal RNA sequences (see 16S RefSeq Nucleotide sequence records) was used as reference database for species assignation of full length 16S sequences. All other data are available in the main text or the supplementary materials.

Research involving human participants, their data, or biological material

Policy information about studies with human participants or human data. See also policy information about sex, gender (identity/presentation), and sexual orientation and race, ethnicity and racism.

Reporting on sex and gender	Both sexes were included
Reporting on race, ethnicity, or other socially relevant groupings	All participants were of Croatian residency and were not asked for ethnicity.
Population characteristics	Six male patients (58,2 ± 18,0, 33 to 81 years old) and 6 females (63 ± 8,1, 56-79 years old) were recruited during July and August 2020 at Split University Hospital. Patients underwent colonoscopy and gastroscopy for gastrointestinal disturbances. Criteria for patient selection were no antibiotic usage one month before sampling and absence of tumors in analysed tissues.
Recruitment	Patients were recruited by clinicians that were not in any other way included in subsequent analysis which is minimizing influence of bias of the study. Potential self-selection is difficult to assess. Selection bias could be present due to having used faecal samples from population with gastrointestinal problems, but authors regard that this does not lessen the value of results obtained with patients' microbiota, as the claims that human microbiota can convert BBN are still valid.
Ethics oversight	The study was approved by the ethical committee of University Hospital of Split (Permit number: 2181-147-01/06/M.S.-20-4) and the University of Split School of Medicine (Permit number: Ur. br. 2181-198-03-04-20-00400) and all participants gave informed consent before participation in the study.

Note that full information on the approval of the study protocol must also be provided in the manuscript.

Field-specific reporting

Please select the one below that is the best fit for your research. If you are not sure, read the appropriate sections before making your selection.

[x] Life sciences [ ] Behavioural & social sciences [ ] Ecological, evolutionary & environmental sciences

For a reference copy of the document with all sections, see nature.com/documents/nr-reporting-summary-flat.pdf

Life sciences study design

All studies must disclose on these points even when the disclosure is negative.

Sample size	No sample size calculation was performed. Instead, the key experiment was repeated five times independently resulting in 30 or more animals per group (Fig. 1b and 1c). In other experiments, sample sizes were kept at ten or less to adhere to 3R standards, but remaining sufficient for statistical comparisons.
Data exclusions	No data was excluded from the analysis.
Replication	Five independent experiments using 20-week exposure BBN or BBN/ABX were performed with in total 30/32 mice in each group respectively. For EHBN experiment, one independent experiment was performed with 8 EHBN/ABX, 9 EHBN treated mice. DBN/PBN experiments using metabolic cages were performed once with 5 mice per group.<br>BBN metabolic cage experiments were repeated twice independently following 3, 6 or 7, and 12 weeks exposure to BBN with 5 animals per group. All attempts at replication were successful.
Randomization	Animals were randomly assigned to study groups. Human and mouse microbiota samples were allocated to all experimental groups as paired samples; therefore, random allocation was irrelevant to this set of measurements.

Blinding	Blinded histological assessment of urinary bladder tissues was performed by researchers and trained pathologists. However, blinding was not performed in any of the other experiments, since in some cases a single researcher was responsible for sacrificing, collecting, and processing samples. When one researcher was collecting while another performed measurements blinding was not planned in advance and thus not conducted. For human and mice microbiota analysis, blinding was not relevant since aim was to identify BBN-converting isolates.

Reporting for specific materials, systems and methods

We require information from authors about some types of materials, experimental systems and methods used in many studies. Here, indicate whether each material, system or method listed is relevant to your study. If you are not sure if a list item applies to your research, read the appropriate section before selecting a response.

Materials & experimental systems

n/a	Involved in the study	
x		Antibodies
	x	Eukaryotic cell lines
x		Palaeontology and archaeology
	x	Animals and other organisms
x		Clinical data
x		Dual use research of concern
x		Plants

Methods

n/a	Involved in the study	
x		ChIP-seq
x		Flow cytometry
x		MRI-based neuroimaging

Eukaryotic cell lines

Policy information about cell lines and Sex and Gender in Research

Cell line source(s)	HEP-G2 (ACC-180) cells were obtained from DSMZ-German Collection of Microorganisms and Cell Cultures GmbH.
Authentication	Cell line was not authenticated.
Mycoplasma contamination	Mycoplasma contamination was tested by PCR reaction on regular basis and used cell line tested negative.
Commonly misidentified lines (See ICLAC register)	Used cell line was not listed as a misidentified in ICLAC registry.

Animals and other research organisms

Policy information about studies involving animals; ARRIVE guidelines recommended for reporting animal research, and Sex and Gender in Research

Laboratory animals	Two-months-old mice c57BL/6J were used in this study.
Wild animals	The study did not involve wild animals.
Reporting on sex	Most experiments were performed on male mice (as bladder cancer is 4 times more common in human males), but in some of them female mice were included (as described in the Methods).
Field-collected samples	The study did not involve samples collected from the field.
Ethics oversight	All mouse experiments were approved by the local IACUC (The University of Split, School of Medicine, Animal Welfare Committee and EMBL IACUC) and the national regulating authorities (Republic of Croatia Ministry of Agriculture, Veterinary and Food Safety Directorate) (permit numbers 525-10/0255-14-4,525-10/0255-15-5, 525-10/0543-21-8, 525-09/566-22-2 and 21-002_HD_MZ).

Note that full information on the approval of the study protocol must also be provided in the manuscript.

Plants

Seed stocks	*Report on the source of all seed stocks or other plant material used. If applicable, state the seed stock centre and catalogue number. If plant specimens were collected from the field, describe the collection location, date and sampling procedures.*
Novel plant genotypes	*Describe the methods by which all novel plant genotypes were produced. This includes those generated by transgenic approaches, gene editing, chemical/radiation-based mutagenesis and hybridization. For transgenic lines, describe the transformation method, the number of independent lines analyzed and the generation upon which experiments were performed. For gene-edited lines, describe the editor used, the endogenous sequence targeted for editing, the targeting guide RNA sequence (if applicable) and how the editor was applied.*
Authentication	*Describe any authentication procedures for each seed stock used or novel genotype generated. Describe any experiments used to assess the effect of a mutation and, where applicable, how potential secondary effects (e.g. second site T-DNA insertions, mosiacism, off-target gene editing) were examined.*
