# Supplementary material for: Gut microbiota carcinogen metabolism causes distal tissue tumours
Source: Nature. 2024 Jul 31;632(8027):1137–44. doi: 10.1038/s41586-024-07754-w (PMC11358042; doi:10.1038/s41586-024-07754-w)
Supplement: Supplementary file 3 — All histology images of mouse bladders after 20 weeks of EHBN exposure. [file 41586_2024_7754_MOESM3_ESM.pdf]

Supplementary Data 2.
All bladder histology images of mouse bladders after 20 week exposure to EHBN chemical. Used for the quantification of cancer stage per mouse summarized in Fig. 5e.

**Treatment**	**Normal**	**CIS**	**Invasion**
EHBN	1	1	7
ABX/EHBN	6		2

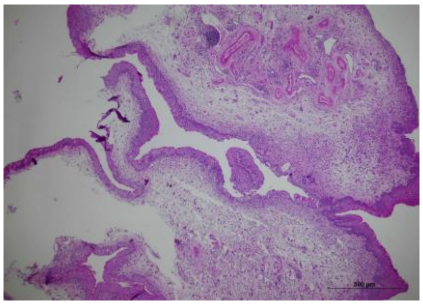
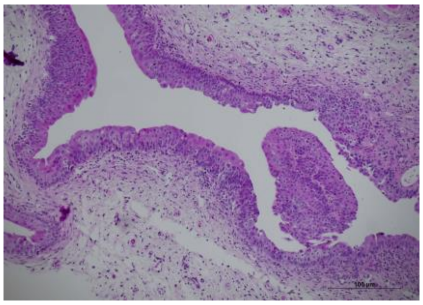
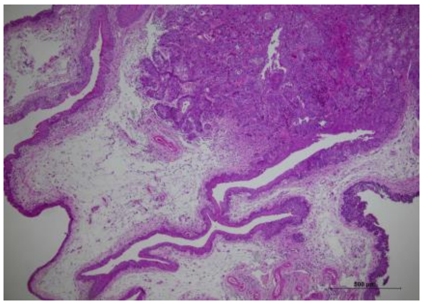
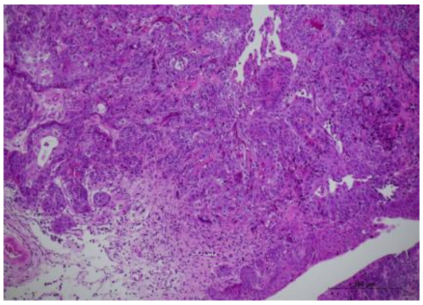
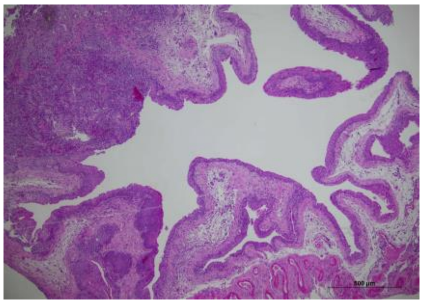
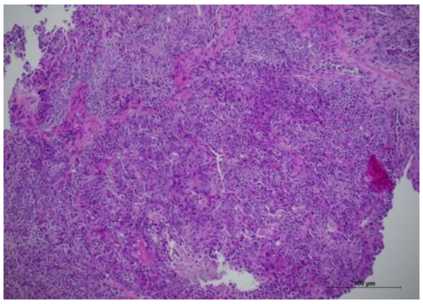
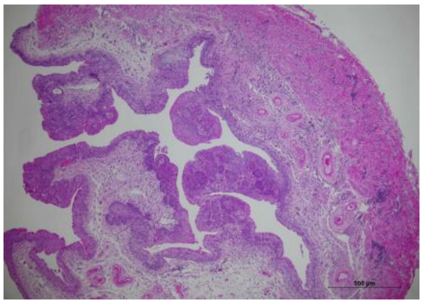
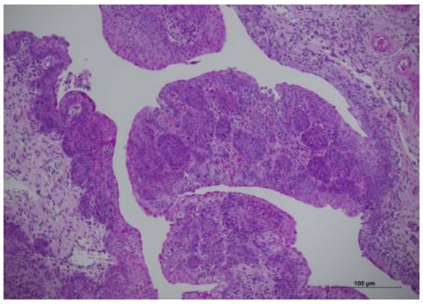
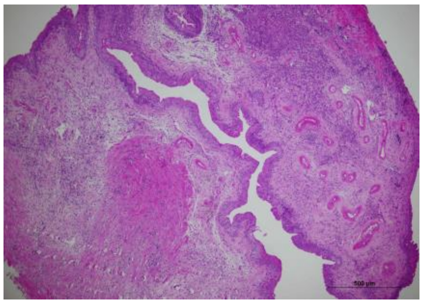
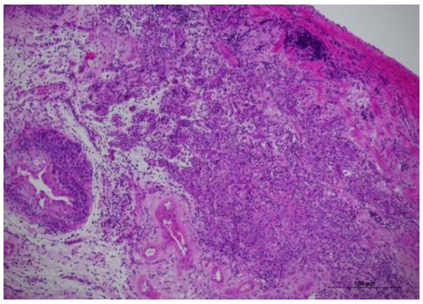

EHBN	1	CIS
EHBN	2	Invasion
EHBN	3	Invasion
EHBN	4	Invasion
EHBN	5	Invasion

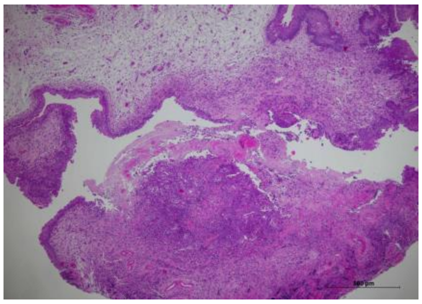
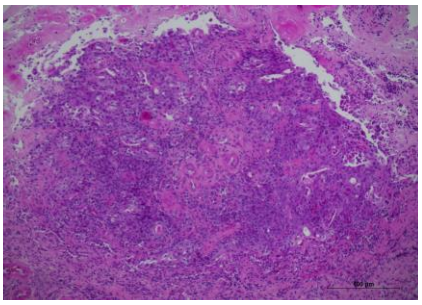

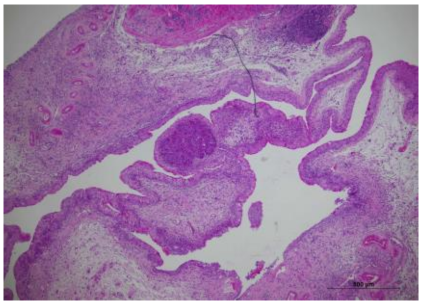
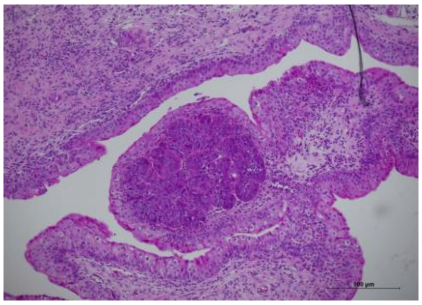

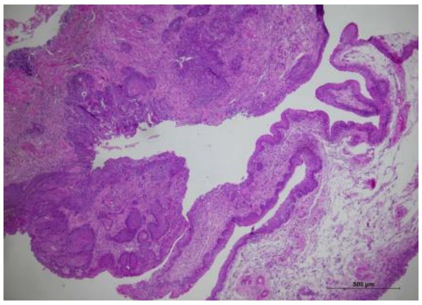
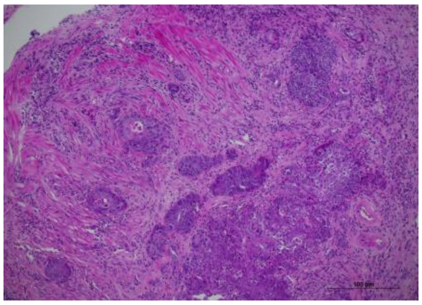

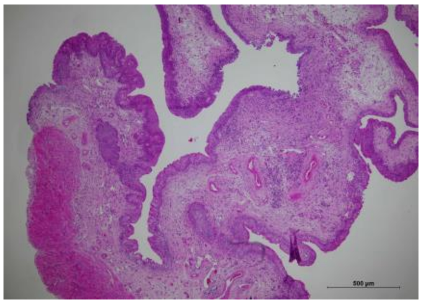
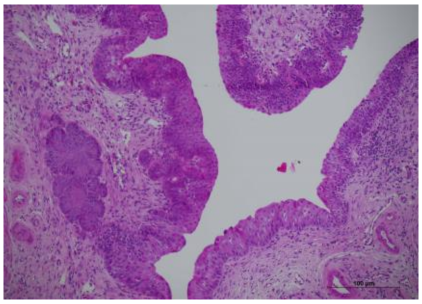

EHBN	6	Invasion
EHBN	7	Invasion
EHBN	8	Invasion
EHBN	9	Healthy

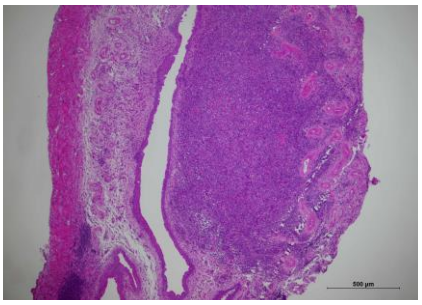
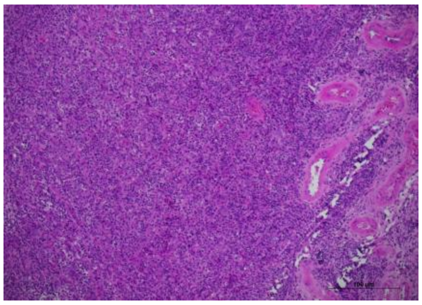

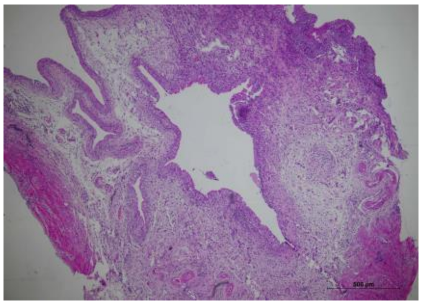
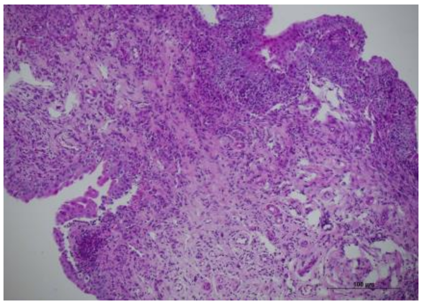

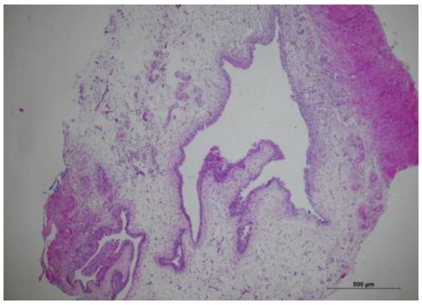
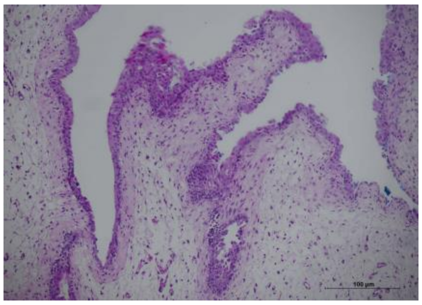

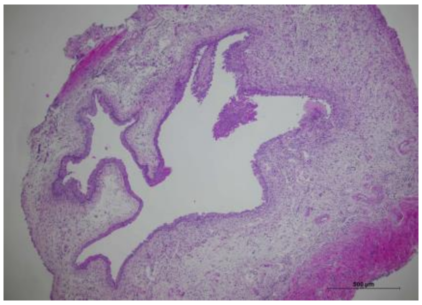
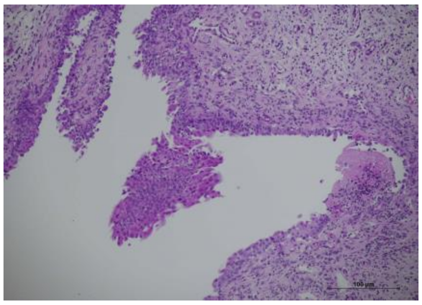

EHBN/ABX	1	Healthy (infammation)
EHBN/ABX	2	Healthy
EHBN/ABX	3	Healthy
EHBN/ABX	4	Healthy

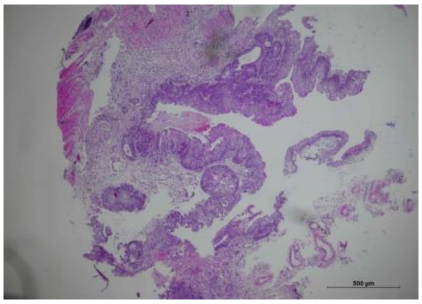	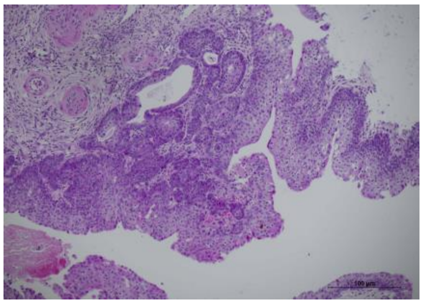	EHBN/ABX	5	Invasion
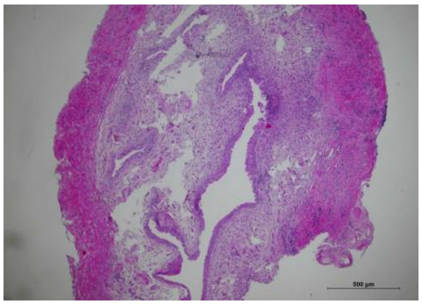	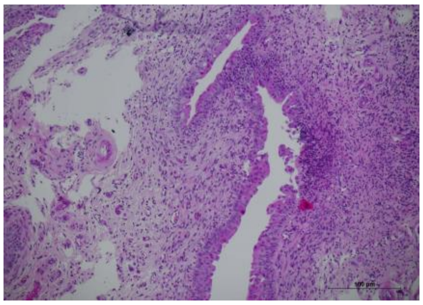	EHBN/ABX	6	Healthy
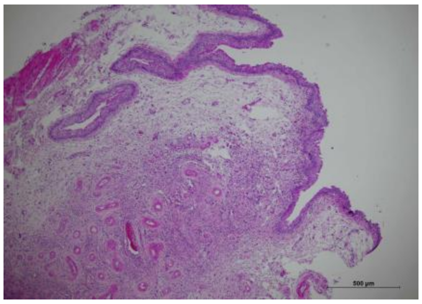	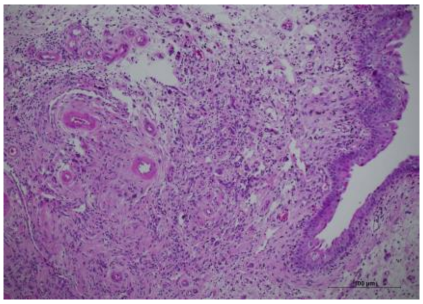	EHBN/ABX	7	Healthy
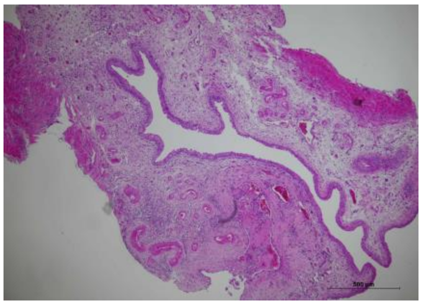	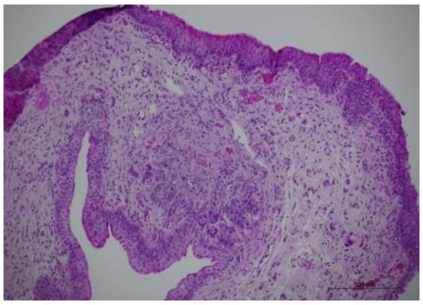	EHBN/ABX	8	Invasion
